# Management of hypercapnic acute respiratory failure with high-flow nasal cannula therapy: A narrative review

**DOI:** 10.1016/j.aicoj.2026.100053

**Published:** 2026-03-24

**Authors:** Christophe Girault, Elise Artaud-Macari, Pierre-Louis Declercq, Jean-Pierre Frat, Jean-Damien Ricard, Arnaud W. Thille, Gaëtan Béduneau

**Affiliations:** a CHU Rouen, Medical Intensive Care Unit, Normandie Univ, UNIROUEN, GRHVN UR 3830, F-76000 Rouen, France; b CHU Rouen, Department of Pulmonary, Thoracic Oncology and Respiratory Intensive Care, Normandie Univ, UNIROUEN, GRHVN UR 3830, F-76000 Rouen, France; c Dieppe Hospital, Medical and Surgical Intensive Care Unit, F-76200 Dieppe, France; d CHU Poitiers, Medical Intensive Care Unit, Poitiers University, INSERM CIC-1402, ALIVE Research Group, F-86000 Poitiers, France; e AP-HP, Medical Intensive Care Unit, Louis Mourier Hospital, Paris Universiy, INSERM, IAME Research Group UMR 1137, F-75000 Paris, France

**Keywords:** Acute respiratory failure, Hypercapnia, Chronic obstructive pulmonary disease, High-flow nasal cannula therapy, Non-invasive ventilation, Extubation

## Abstract

Hypercapnic acute respiratory failure (ARF) is a life-threatning condition and a leading cause of hospital admission, mainly in acute exacerbation of chronic obstructive pulmonary disease (COPD). Although non-invasive ventilation (NIV) currently represents the first-line ventilatory strategy in patients exhibiting hypercapnic ARF, NIV may fail primarily due to worsening of ARF, patient-ventilator asynchrony, interface intolerance, even cardiovascular instability. Consequently, the operating principles and physiological effects of HFNC could be interesting and useful for applying this technique to the management of hypercapnic ARF. Therefore, from a clinical point of view, HFNC could be used alone continuously as an alternative to standard oxygen therapy or NIV, either at the initial phase of hypercapnic ARF or after extubation. In addition, according to the severity and etiology of the underlying hypercapnic ARF, HFNC could also be used in combination with NIV during breaks of spontaneous breathing. In this narrative review, we aimed therefore to provide an updated overview of the physiological rationale and clinical evidence for using HFNC in patients with hypercapnic ARF, with a particular focus on acute exacerbations of COPD.

## Introduction

Conventional oxygen therapy is the gold standard for the symptomatic treatment of all acute respiratory failure (ARF), whether hypoxemic or hypercapnic in origin. Since the original publication of the « FLORALI » study by Frat et al. [1] 10 years ago, high-flow humidified and warmed nasal cannula (HFNC) therapy has continued to develop, becoming the first-line treatment for hypoxemic ARF [2,3]. Since the global SARS-CoV2 pandemic, the use of HFNC has grown considerably in critical care units, respiratory intensive care units (RICUs), emergency departments, and even conventional pulmonary medicine wards [4]. The technical characteristics and operating principles of HFNC confer numerous beneficial physiological effects, improving not only oxygenation conditions but also the breathing pattern and mechanics of patients [5,6]. In fact, due to its interdependent physiological effects, which notably reduce respiratory effort, HFNC should no longer be considered as a simple oxygenation strategy, but also as a real mode of respiratory support which is well tolerated [7]. Although mainly evaluated in hypoxemic ARF [[5], [6], [7]], the physiological effects of HFNC and its clinical use could nevertheless prove useful in the management of hypercapnic ARF [8].

We aimed, therefore, to conduct a narrative review to provide an updated overview of the physiological rationale and clinical evidence for using HFNC in patients with hypercapnic ARF with a particular focus on acute exacerbations of chronic obstructive pulmonary disease (AE-COPD). This narrative review was based on a systematic literature search via PubMed for relevant English-language studies from 2015 to 2025. Using the following keywords, « HFNC », «hypercapnic ARF », « hypercapnia », « COPD », « AE-COPD », « non invasive ventilation (NIV)», « post-extubation period », study selection included any observational studies, randomized controlled trials, systematic review and/or meta-analysis involving adults patients.

## Operating and physiological rationale of high-flow nasal cannula therapy during hypercapnic acute respiratory failure

### Operating principles and main physiological effects of high-flow nasal cannula therapy

The main operating principles of HFNC are based on the ability to deliver humidified and warmed oxygen (warmed to 37 °C and actively humidified: 44 mg of H_2_O/L, i.e., 100% relative humidity) with a controlled and adjustable fraction of inspired oxygen (FiO_2_) of 21 to 100%, at a set gas flow rate of up to 70 L/min depending on the device used (specific column or ventilator), so as to cover the peak inspiratory flow rate of patients in acute respiratory distress [8]. These operating principles give rise to a number of physiological effects, which are responsible for the beneficial effects of HFNC initially described in hypoxemic ARF [[5], [6], [7]]. These physiological effects include: positive end-expiratory pressure (PEEP), which is more pronounced (3–5 cmH_2_O) when the patient breathes with a closed mouth; continuous wash-out of the anatomical dead space in the upper airways by the flow of fresh gas delivered, increasing carbon dioxide (CO_2_) clearance by limiting or even reducing its re-inhalation; decreased airway resistance and improved mucociliary clearance due to the quality of active humidification; and improved patient breathing pattern (decreased respiratory rate, maintenance of tidal volume, decreased minute ventilation) without worsening alveolar ventilation (pH, partial arterial pressure of carbon dioxide (PaCO_2_), in particular through a reduction in respiratory effort, inspiratory resistance, an increase in alveolar recruitment, an improvement in dynamic lung compliance, and a more homogeneous distribution of ventilation within the lung parenchyma. Clinicians should also bear in mind that during HFNC, its various physiological effects, the accuracy of the administered FiO_2_, and its benefit on signs of ARF appear to be closely correlated with the amount of gas flow applied [9]. Finally, the operating principles and physiological effects described above are highly interdependent in order to ultimately optimize oxygenation and respiratory comfort, improve patients' breathing pattern and ventilatory mechanics, while reducing their respiratory effort [6] In fact, in addition to the physiological effects described above, the benefits of HFNC during ARF may be due to other mechanisms. Indeed, it has recently been shown that, in orally intubated patients, « nasal » HFNC can significantly reduce dyspnea and respiratory center activity, independently of any decrease in respiratory effort and/or improvement in oxygenation [10]. These data therefore suggest an additional and very interesting physiological effect of HFNC to decrease respiratory drive, notably by restoring nasal breathing, which is logically suppressed in intubated patients.

Finally, although these physiological data have been demonstrated mainly in hypoxemic ARF [[5], [6], [7],^9^], they could also prove useful in other indications, particularly in the management of hypercapnic ARF.

### Physiological rationale for applying high-flow nasal cannula therapy to hypercapnic acute respiratory failure

In addition to the physiological benefits of NIV during hypercapnic ARF [11], the physiological effects of HFNC, as outlined above, suggest that this technique may have a potentially beneficial effect during hypercapnic ARF, particularly in the management of AE-COPD (Table 1). It should be noted, however, that in COPD patients we currently have more physiological data available for patients in a stable condition rather than for those in acute hypercapnic decompensation.

**Table 1:** Main physiological rationale for using HFNC in hypercapnic ARF.

**Contribution of high gas flow rate**
Wash-out of the nasopharyngeal anatomical dead space
Anti-rebreathing effect (CO_2_)
Improved breathing pattern (↓ RR,↑ TV,↓ VE)
Improved alveolar ventilation (pH, PaCO_2_)
Moderate extrinsic PEEP effect (3–5 cmH_2_O)
↓ (counterbalance) intrinsic-PEEP
↑ initiation of inspiratory effort
Improved ventilatory mechanics
↓ work of breathing
Reduced upper airways resistance
**Optimization of oxygenation**
Accuracy of FiO_2_ control in the presence of hypercapnia
**Humidification and warming of inhaled gases**
Preservation of mucociliary clearance
Fluidification of bronchial secretions (COPD)
Respiratory comfort

HFNC: high-flow nasal cannula; ARF: acute respiratory failure; RR: respratory rate; TV: tidal volume; VE: minute ventilation; PEEP: positive end expiratory pressure; COPD: chronic obstructive pulmonary disease.

#### Anatomical dead space wash-out

Continuous wash-out of the nasopharyngeal dead space, by supplying a continuous flow of fresh gas, is one of the most relevant physiological effects supporting the use of HFNC in hypercapnic ARF.

Indeed, the supply of a high gas flow rate can help to limit the re-inhalation of CO_2_ from exhaled gas and thus increase CO_2_ clearance [12]. This interesting aspect in hypercapnic ARF was initially suggested by the absence of any significant worsening of PaCO2 and/or pH on HFNC in the first clinical-physiological studies conducted in hypoxemic ARF [2,13]. Subsequently, several studies have suggested a potential physiological benefit of HFNC in patients with stable COPD, namely an improvement in breathing pattern (decrease in respiratory rate, maintenance or increase in tidal volume, decrease in minute ventilation) [14,15], a reduction in respiratory effort [16,17], and an improvement in alveolar ventilation (pH, PaCO_2_) [18,19]. In COPD patients, HFNC has also been shown to reduce central ventilatory control activity following extubation [20] and to decrease respiratory effort after weaning from NIV [21]. These physiological effects, observed in COPD patients, appear to be closely linked to the continuous wash-out effect of CO_2_ from the anatomical dead space [16]. Nevertheless, the CO_2_ wash-out being non-linearly correlated with the HFNC flow rate [9] it also appears related to the level of occlusion of the nostrils, and therefore to the level of leakage generated around the nasal cannulas [18]. In COPD patients, a higher HFNC flow rate not only increased the pressure in the upper airways but also helped to improve CO_2_ clearance, particularly in cases of high respiratory rate (ARF) [22]. One of the physiological studies by Bräunlich et al. [19] also suggested that the effect on lowering PaCO_2_ could be similar to that observed with NIV in stable COPD patients. More recently, a prospective cross-over physiological study confirmed that, compared to conventional oxygen therapy, HFNC at 40 L/min significantly improved the breathing pattern in 10 patients following AE-COPD, in particular by reducing dead space as reflected by the decrease in the ventilatory ratio [23]. Finally, the wash-out of the upper airways reduces dead space, which in turn reduces minute ventilation and respiratory rate. This can contribute therefore to a very interesting physiological benefit in COPD patients by reducing the CO_2_ production, dynamic hyperinflation and intrinsic PEEP, as well as inspiratory effort [[14], [15], [16]].

The anti-rebreathing effect of CO_2_ appears also to be further optimized depending on the design of the HFNC cannulas used. In a test bench study, it was demonstrated that, compared to standard symmetrical cannulas, asymmetrical cannulas significantly increased CO_2_ clearance and positive pressure in the upper airways [22,24]. The benefit of asymmetric cannulas on CO_2_ clearance was found to be greater when the respiratory rate and gas flow rate were high, particularly in cases of obstructive profiles such as COPD [24]. The authors attributed these beneficial physiological effects to the fact that asymmetric HFNC cannulas generate reverse flow between the nasal cavities, particularly via the choanae, thereby accelerating CO_2_ clearance through the less occluded nostril [24]. A reduction in minute ventilation and respiratory work was also reported with asymmetric cannulas compared to standard cannulas in 10 patients with moderate hypoxemic ARF [25]. However, such a physiological benefit has yet to be demonstrated in cases of AE-COPD, especially since the size of the cannulas appears to have little influence on the effect of HFNC in COPD patients, at least in stable condition [26].

However, initial experimental results in terms of dead space clearance and pressure generated in the upper airways [22,24] suggest that the physiological benefits of asymmetric cannulas during HFNC could prove to be even more significant in cases of hypercapnic ARF. In the meantime, while the effects of HFNC appear to be closely correlated with the gas flow rate applied [9], excessively high flow rates, particularly those exceeding the patient's instantaneous inspiratory flow rate, expose the patient to a risk of pulmonary overdistension and respiratory discomfort [27]. It should also be noted that a recent prospective physiological study including 20 patients recovering from AE-COPD did not show any additional benefit of a HFNC flow rate > 40 L/min in terms of comfort and reduction of respiratory effort [28].

All of these data should therefore encourage clinicians to individualize HFNC settings, in particular by titrating the gas flow rate for a given patient, especially in COPD patients. Indeed, the gas flow rate titration must above all take into account its clinical tolerance in terms of respiratory comfort, as a too high flow rate (> 40 L/min) could be harmful in these patients, potentially aggravating their pulmonary hyperinflation by excessively increasing expiratory resistance [18,28].

#### Positive end-expiratory pressure effect and reduction in airway resistance

Initially demonstrated in the nasopharyngeal area in healthy volunteers [29], the possibility of generating a PEEP effect on HFNC was subsequently demonstrated by electrical impedance tomography at the distal airways level [7,30,31]. The PEEP effect, reflecting the potential ability of HFNC to generate a certain level of alveolar recruitment, results from the expiratory resistance produced by the cannulas during patient exhalation. However, this PEEP effect appears relatively moderate (3–5 cmH_2_O) [7,[29], [30], [31]] and varies depending on whether the mouth is open or closed, the flow rate applied, and the size of the cannulas in relation to that of nostrils [7,22,29]. Due to a greater increase in pressure in the upper airways with asymmetric cannulas, these could also be likely to generate, at comparable settings, an even greater PEEP effect than with symmetrical cannulas [22,24]. Finally, as with NIV [11,32], it is therefore conceivable that the PEEP effect with HFNC may help to partially counterbalance the intrinsic PEEP observed in many COPD patients, especially those with decompensation, thus facilitating exhalation, decreasing the risk of dynamic pulmonary hyperinflation and, ultimately, reducing respiratory effort both experimentally [7] and in stable conditions [16,17] or in acute situations [20,21]. By generating high inspiratory flow rates, HFNC also mechanically counterbalances pharyngeal collapse, which is commonly observed during negative pressure inspiration, by maintaining positive pharyngeal pressure and thus reducing airway resistance [8,33]. The ability to counterbalance pharyngeal collapse during inspiratory effort may also partly explain the reduction in respiratory effort observed with HFNC [5,7]. The decrease in airway resistance can only be enhanced by better conditioning (humidification and warming) of the inspired gases on HFNC, as opposed to dry, cold gas on standard oxygen therapy [34]. Such a reduction in airway resistance could therefore prove potentially beneficial during hypercapnic ARF in COPD patients.

#### Optimization of oxygenation

One of the operating principles of HFNC is its ability to generate high flow rates of an air-O_2_ mixture (20–70 L/min depending on the device), and therefore to reach or exceed the patient's instantaneous inspiratory flow rate in cases of acute respiratory distress, which can exceed 60 L/min during acute exacerbations of COPD [35]. By covering the peak flow rate and reducing the dilution of FiO_2_ by ambient air, HFNC, unlike standard oxygen therapy (nasal cannula or high-concentration mask), can not only meet the patient's ventilatory need but also deliver a stable FiO_2_ corresponding to the prescribed level, which can ultimately be controlled from 21 to 100% [8]. The actual FiO_2_ inspired was found to be all the more reliable when a high gas flow rate was used (> 30 L/min) [27,36].

In addition to the PEEP effect, it is likely that the wash-out of the anatomical dead space during exhalation, described above, may also play a significant role in optimizing oxygenation on HFNC. Indeed, during inspiration, the initial amount of inspired gas is more enriched in oxygen due to the effect of HFNC, unlike the gas that is normally depleted in oxygen during physiological expiration [12]. However, while the benefits of HFNC on oxygenation appear to be greater than those of conventional oxygen therapy, this does not seem to be the case when compared to NIV, probably due to the possibility of applying a higher level of external PEEP with NIV [11,37]. Nevertheless, such precise control of FiO_2_ on HFNC may be relevant in patients at risk of hypercapnia induced by standard oxygen therapy, such as during AE-COPD [38], provided that the gas flow rate is correctly titrated according to the FiO_2_ with HFNC [39].

#### Gas conditioning and respiratory comfort

The gas conditioning of HFNC described above in terms of warming and humidification not only allows for high gas flow rates (up to 70 L/min depending on the device) but also helps to preserve mucociliary clearance, thereby facilitating the expectoration of bronchial secretions [8,40]. This “bronchial fluidifying” effect, demonstrated in patients with bronchiectasis [41], can therefore be beneficial in patients with decompensated COPD [42]. Compared to standard oxygen therapy, the improved gas conditioning provided by HFNC also improves respiratory comfort in terms of pharyngeal pain and dryness of the upper airways [43]. Finally, compared to NIV masks, HFNC, which uses simple flexible silicone nasal cannulas, appears more comfortable and better tolerated, making it easier for patients to speak, eat, and cough up secretions in acute situations [21,37]. In addition to the various factors that can influence humidity with HFNC (patient, environment, settings) [44], the feeling of comfort on HFNC seems to be influenced more by the temperature level than by the flow rate applied [45]. Finally, it has recently been demonstrated that, regardless of oxygenation levels and the effect on ventilatory mechanics, HFNC can also significantly improve dyspnea in healthy subjects or patients, possibly by simply restoring nasal breathing [10,46]. These data on humidification quality and respiratory comfort with HFNC are therefore potentially usefull in cases of hypercapnic ARF during AE-COPD.

## Clinical application of high-flow nasal cannula therapy in hypercapnic acute respiratory failure

Although NIV remains the first-line ventilatory strategy in the management of hypercapnic ARF, and more specifically in AE-COPD [47], the physiological aspects discussed above suggest that HFNC could be effectively applied to hypercapnic ARF. Furthermore, clinicians must also take into account the fact that NIV exposes patients to patient-ventilator asynchrony, which is sometimes difficult to avoid despite optimized settings [48], as well as to a certain degree of clinical discomfort and therefore a risk of intolerance to the technique, both of which can lead to refusal and/or failure of NIV [48,49].

Initially, several case reports illustrated the potential benefits of HFNC in hypercapnic ARF in patients who had failed standard oxygen therapy and/or NIV [[50], [51], [52]]. Subsequently, several clinical-physiological or clinical studies have investigated the potential role of HFNC in the management of hypercapnic ARF in comparison with conventional oxygen therapy, NIV, or associated with the latter.

### High-flow nasal cannula therapy as an alternative to standard oxygen therapy

The first interesting results come from observational cohort studies, retrospective or prospective, mainly including patients with mild to moderate COPD [[53], [54], [55], [56]] (Table 2). These studies have essentially shown that, compared to standard oxygen, HFNC significantly reduces PaCO_2_, prevents worsening or even corrects more or less severe respiratory acidosis, decreases respiratory rate, and is well tolerated. The arterial blood gas benefit in terms of alveolar ventilation (pH, PaCO_2_) was greater in patients who were more hypercapnic at baseline [53,54]. In the retrospective study by Kim et al. [55], which included 33 patients with hypercapnic ARF, a decrease in PaCO_2_ was observed within the first hour of HFNC use as first-line treatment at a flow rate between 40 and 50 L/min. In a randomized cross-over study comparing HFNC and standard oxygen therapy, Pilcher et al. [57] confirmed the decrease in transcutaneous partial pressure of CO_2_ (PtcCO_2_) on HFNC at 35 L/min as early as the thirtieth minute in 24 patients with AE-COPD. A prospective single-center study also showed that, in 15 COPD patients with associated bronchiectasis and mild exacerbation (pH ≥ 7.35), HFNC at 50 L/min not only improved hematosis (PaO_2_, PaCO_2_), respiratory rate, and dyspnea in patients during the first 72 h of evaluation, but also increased mucus production and their ability to expectorate [58]. However, it has been suggested that the decrease in PaCO_2_ on HFNC during the first 24 h may be more significant in moderately hypercapnic patients without underlying obstructive respiratory disease [59]. The first prospective, randomized, multicenter study in this indication, including 320 patients with mild to moderate AE-COPD (pH ≥ 7.35, PaCO_2_ > 45 mmHg), not only confirmed the efficacy of HFNC (n = 160) in reducing PaCO_2_, but also demonstrated a lower risk of invasive or non-invasive mechanical ventilation compared to patients receiving standard oxygen therapy alone (n = 160) [60] (Table 2). However, the benefit in terms of risk of intubation and technical failure was not confirmed in a second randomized multicenter study comparing these two oxygenation strategies in 330 patients with hypercapnic AE-COPD with equally mild to moderate severity without acidosis [61]. There was no difference in survival between the two strategies, but HFNC increased length of stay and hospital costs. The authors explained these negative results by a potential lack of power of the statistical analysis of the study with regard to the primary endpoint (need for intubation), which was found to be lower (≤ 2.5%) than initially expected in both groups [61]. In an ancillary study, the same team confirmed the longer length of stay with HFNC in these same patients, especially in those with high baseline bicarbonate levels (≥ 35 mmol/L) [62]. Despite these somewhat conflicting clinical data, recent systematic reviews and/or meta-analyses incorporating previous randomized studies consider that, compared to conventional oxygen therapy, HFNC improves gas exchange (reduction in PaCO_2_) and reduces the risk of treatment failure, particularly the use of NIV, in COPD patients with mild acute hypercapnic decompensation (without acidosis: pH ≥ 7.35) [[63], [64], [65]] (Table 3).

**Table 2:** Main observationnal and randomized clinical studies using HFNC as an alternative to standard oxygen therapy in acute hypercapnic ARF.

Authors [reference] year	Country	Study design (global population)	Location	Study group (s) (n patients)	Initial HFNC settings	End-points (*primary)	Main results (with HFNC vs O_2_)
Jeong et al. [53] 2015	Korea	Single center Retrospective Observational (n = 81)	Emergency department	Hypercapnic ARF PaCO_2_ > 45 mmHg (n = 46) Non hypercapnic ARF (n = 35)	Flow = 42 ± 14 L/min FiO_2_ = 48 1.6% Flow = 49 ± 11 L/min FiO_2_ = 66 ± 0.1%	. Changes in ABG/baseline (O_2_)* . Worsening to NIV and IMV . Mortality	. ↓ PaCO_2_ in both groups . Only significant in the hypercapnic group (from73.2 ± 20.0 to 67.2 ± 23.4 mmHg; p = 0.02) . Similar worsening to NIV, IMV and mortality
Bräunlich et al. [54] 2018	Germany	Single center Retrospective Observational (n = 38)	Respiratory ward	Hypercapnic AE-COPD pH ≤ 7.38, PaCO_2_ > 45 mmHg (subgroup pH < 7.35, n = 17)	Flow = 26 ± 8 L/min FiO_2_ for baseline SpO_2_	. Changes in ABG/baseline (O_2_)*	. Significant ↑ in pH (7.33 ± 0.04 to 7.39 ± 0.04; p = 0.000) and ↓ in PaCO_2_ (67.6 ± 12.9 to 58.5 ± 9.7; p = 0.001) . Greater ABG improvement in more acidotic patients (pH < 7.35)
Kim et al. [55] 2018	Korea	Single center Retrospective Observational (n = 33)	ICU	Hypercapnic ARF PaCO_2_ > 45 mmHg	Flow = 41 ± 1 L/min FiO_2_ = 45 ± 0.2%	. Changes in ABG/baseline (O_2_)*	. Significant ↓ in PaCO_2_ after 1 h (55.5 to 52.7 mmHg; p = 0.006) and 24 h (↓ by 3.7 ± 10.8 mmHg; p = 0.062) . No change in pH
Plotnikow et al. [56] 2021	Argentina	Multicenter Prospective Observational (n = 40)	5 ICUs	Hypercapnic AE-COPD (pH < 7.35, PaCO_2_ > 50 mmHg (severe 62.5%)	Flow = 40 L/min FiO_2_ for SpO_2_ ≥ 92%	. Changes in RR and ABG after 1 h/baseline (O_2_)*	. Significant ↓ in RR (p < 0.001) and PaCO_2_ (57 to 52 mmHg; p < 0.001) . No significant change in RR, pH and PaCO_2_ during the first hour in 18% of patients who failed HFNC
Pilcher et al. [57] 2017	New-Zealand	Single center Prospective Randomized Cross-over (n = 24)	Medicine ward	Hypercapnic AE-COPD HFNO followed by O_2_ (n = 12) O_2_ followed by HFNO (n = 12)	Flow = 35 L/min FiO_2_ for SpO_2_ ≥ 94%	. Changes in PtCO_2_/baseline (O_2_)*	. Significant ↓ in PtCO_2_ within the first 30 min (p = 0.001)
Crimi et al. [58] 2020	Italy	Single center Prospective Observational (n = 15)	Respiratory ward	Hypercapnic AE-COPD and bronchiectasis (pH ≥ 7.35, PaCO_2_ > 50 mmHg)	Flow = 50 L/min FiO_2_ for SpO_2_ ≥ 92%	. Changes in ABG and respiratory parameters after 24 h/baseline (O_2_) * . Mucus production . HFNC tolerance	. Significant ↓ in dyspnea score (p < 0.001), RR (p < 0.001) and PaCO_2_ (p = 0.003) . Significant ↑ in mucus production . Good HFNC tolerance
Nam et al. [59] 2021	Korea	Multicenter Prospective Observational (n = 45)	ED General ward ICU	ARF with mild to moderate hypercapnia (43 ≤ PaCO_2_ ≤70 mm Hg) Obstructive (n = 18) vs non obstructive patients (n = 24)	Flow = 35 L/min FiO_2_ for SpO_2_ ≥ 92%	. Changes in ABG after 24 h / baseline (O_2_) * . Changes in RR . Worsening to NIV or IMV	. Significant ↓ in PaCO_2_ (p < 0.001) and ↑ in pH (p < 0.001) as early 1rst hour . ↓ in PaCO_2_ greater in non obstructive patients . No significant change in RR and no worsening for 42/45 pts (93.3%)
Li et al. [60] 2020	China	Multicenter Prospective Randomized (n = 320)	Respiratory ward	Mild to moderate AE-COPD (pH ≥ 7.35, PaCO_2_ > 45 mmHg) HFNC (n = 160) vs O_2_ (n = 160)	Flow = 33 ± 6 L/min FiO_2_ for SpO_2_ 90−93% Standard O_2_ = 2−4 L/min for SpO_2_ 90−93%	. Treament failure (NIV and/or IMV) * . Changes in ABG and RR at 24 h . Subjective discomfort score,LOS	. Lower failure rate (p = 0.026) . Significant ↓ in PaCO2 (p = 0.030) and RR (p = 0.024) . Significant ↓ in discomfort at day 5 (p < 0.001) . No significant difference in LOS
Xia et al. [61] 2022	China	Multicenter Prospective Randomized (n = 330)	Respiratory ward	Mild AE-COPD (pH ≥ 7.35, PaCO_2_ > 45 mmHg) HFNC (n = 158) vs O_2_ (n = 172)	Flow: 25–40 L/min FiO_2_ for SpO_2_ 90−95% Standard O_2_ = 1–5 L/min for SpO_2_ 90−95%	. Need (criteria) for intubation* . Treament failure (intolerance and need for NIV and/or IMV) . Mortality, LOS, hospital cost, readmission at day 90	. No significant difference in the need for intubation, treatment failure, mortality and readmission at day 90 . Longer LOS (p = 0.021 ) and higher hospital cost (p = 0.006)

HFNC: high-flow nasal cannula; ARF: acute respiratory failure; AE-COPD: acute exacerbation of chronic obstructive pulmonary disease; NIV: non invasive ventilation; IMV: invasive mechanical ventilation; ICU: intensive care unit; ED: emergency department; ABG: arterial blood gas; RR: respiratory rate; PtCO_2_: transcutaneous carbon dioxide tension; LOS: length of stay.

**Table 3:** Systematic review and meta-analysis assessing HFNC in acute hypercapnic ARF.

Authors [reference] year	Country	Study design selected	Studies included (n)	Global population (n patients/group)	End-points (*primary)	Main results
**HFNO as an alternative to standard oxygen therapy**						**With HFNC vs O_2_**
Zhang et al. [63] 2023	China	RCTs and cross-over studies (n = 8)	5/8 studies in hypercapnic ARF	810 patients HFNC (n = 397) vs O_2_ (n = 413)	• Treatment failure (worsening to NIV) * • Changes in ABG and RR	• Significant ↓ in treatment failure and PaCO_2_ • No significant difference in PaO_2_ and RR
Yang et al. [64] 2023	China	RCTs and cross-over studies (n = 31)	2/31 studies in AE-COPD	650 patients HFNC (n = 318) vs O_2_ (n = 332)	• Need for intubation* • Treatment failure (intubation and/or switch to NIV) • Changes in ABG • ICU and hospital LOS, readmission	• No significant difference in intubation rate • Significant ↓ in treatment failure and PaCO_2_ • No significant difference in hospital LOS or readmission
Xu et al. [65] 2023	China	RCTs (n = 10)	2/10 studies in hypercapnic ARF	650 patients HFNC (n = 318) vs O_2_ (n = 332)	• Need for intubation* • Treatment failure (intubation and/or switch to NIV) • Changes in ABG • Hospital LOS, mortality	• No significant difference in intubation rate • Significant ↓ in treatment failure • No significant difference in ABG changes, hospital LOS and mortality
**HFNO as an alternative to non invasive ventilation**						**With HFNC vs NIV**
Pisani et al. [79] 2019	Italy	RCTs and cross-over studies (n = 5)	3/5 studies in AE-COPD	190 patients HFNC (n = 96) vs NIV (n = 94)	• Changes in ABG* • Changes in RR, WOB and comfort • Outcome (intubation, mortality)	• Significant ↓ or no change in PaCO_2_, • Similar ↓ in RR and WOB, • Better comfort • No significant difference in outcome
Ovtcharenko et al. [80] 2022	Canada	RCTs and cross-over studies (n = 8)	8 studies in hypercapnic ARF	528 patients HFNC (n = 275) vs NIV (n = 253)	• Mortality rate* • Need for intubation • Changes in ABG (PaCO_2_) • Hospital LOS	• No significant difference in mortality, intubation rate and hospital LOS • No significant difference in ABG improvement
Yang et al. [64] 2023	China	RCTs and cross-over studies (n = 31)	2/31 studies in AE-COPD (non post-extubation)	122 patients HFNC (n = 63) vs NIV (n = 59)	• Need for intubation* • Treatment failure (intubation and/or treatment switch to other group) • Changes in ABG • ICU and hospital LOS, readmission	• No significant difference in intubation rate, treatment failure, ICU and hospital LOS, mortality and ABG improvement • More switch in more severe hypercapnic ARF (pH < 7.30)
Xu et al. [65] 2023	China	RCTs (n = 10)	5/10 studies in hypercapnic ARF (non post-extubation)	395 patients HFNC (n = 201) vs NIV (n = 194)	Need for intubation* Treatment failure (intubation and/or treatment switch to other group) Changes in ABG Hospital LOS, mortality	• No significant difference in intubation rate, treatment failure, mortality and ABG improvement • Better comfort and lower complications • ↓ in hospital LOS • More switch in more severe hypercapnic ARF (pH < 7.30)
Fahey et al. [81] 2023	Ireland	RCTs (n = 7)	7 studies in hypercapnic ARF	471 patients HFNC (n = 236) vs NIV (n = 235)	Changes in ABG* (PaCO_2_, pH, PaO_2_) Intubation rate Treatment switch to other group Mortality	• No significant difference in ABG changes • No significant difference in intubation rate switch and mortality
Qin et al. [85] 2025	China	RCTs (n = 4)	4 studies in AE-COPD	486 patients HFNC (n = 239) vs NIV (n = 247)	• Mortality • Need for intubation • Treatment failure (intubation and/or treatment switch to other group) • Treatment switch	• No significant difference in mortality and intubation rate • ↑ the risk of treatment failure with more patients requiring a treatment switch

HFNC: high-flow nasal cannula; ARF: acute respiratory failure; AE-COPD: acute exacerbation of chronic obstructive pulmonary disease; NIV: non invasive ventilation; RCT: randomized controlled trial; ICU: intensive care unit; ED: emergency department; ABG: arterial blood gas; RR: respiratory rate; WOB: work of breathing; LOS: length of stay.

Finally, it should be noted that following severe COPD exacerbations in the recovery phase, HFNC applied in a very early rehabilitation program may improve exercise tolerance while reducing systemic inflammation, compared to standard oxygen therapy [66]. Interestingly, beyond the acute phase of hypercapnic ARF and its rehabilitation, it has recently been shown that HFNC applied at home in severe stabilized COPD patients, compared to long-term oxygen therapy, not only improves dyspnea, 6 min walking test, PaCO_2_, and quality of life, it can also reduces the rate of acute exacerbations and hospitalizations in these patients [67,68].

### High-flow nasal cannula therapy as an alternative or in combination with non-invasive ventilation

#### Initial management of hypercapnic acute respiratory failure

##### High-flow nasal oxygen therapy as an alternative to non-invasive ventilation

In the clinical cases discussed above, the use of HFNC in COPD patients with acute hypercapnic decompensation was mainly motivated by patients’ intolerance to the NIV mask [50,52] or the existence of significant leaks that were difficult to control [51]. In all reported cases, a benefit of HFNC was observed, both in terms of blood gas values and tolerance of the technique [[51], [52], [53], [54]].

In an initial retrospective cohort of 82 moderate COPD patients (pH = 7.25–7.35; PaCO_2_ > 50 mmHg), no difference was found in PaCO2 correction, treatment failure (intubation or strategy switch), or mortality at D28 when comparing HFNC (n = 39) to NIV (n = 43) [69] (Table 4). However, the authors reported that HFNC could be used for longer with fewer interventions by the healthcare team than NIV during the first 24 h, with NIV also causing more skin lesions during treatment. However, another retrospective cohort study, evaluating a resuscitation database of patients with mild hypercapnia at admission (45 < PaCO_2_ < 60 mmHg), did not confirm these positive results [670]. On the contrary, the propensity score-matched analysis revealed that HFNC (n = 106) was less beneficial than NIV (n = 106), increasing the use of intubation and mortality at D28, as well as the length of stay in ICU. Subsequently, a prospective randomized cross-over physiological and clinical feasibility study suggested that HFNC and NIV were equally effective in improving, in the very short term (30 min), oxygenation and signs of respiratory distress in 30 COPD patients with moderately severe acidosis (7.25 < pH < 7.35; PaCO_2_ ≥ 45 mmHg) [71]. The latest published retrospective cohort study, including 100 COPD patients with moderately severe hypercapnic ARF (7.30 < pH < 7.35; PaCO_2_ ≥ 58 mmHg), also showed equivalent rates of failure (intubation, switch, or death), length of stay, and mortality at day 28 for HFNC (n = 46) compared to NIV (n = 54) [72]. Non-invasive ventilation improved respiratory parameters (respiratory rate, PaCO_2_) more rapidly but was much less well tolerated than HFNC, whose failure was, however, associated with worsening of respiratory distress and/or hypercapnia. In a prospective study randomizing 88 moderately severe hypercapnic COPD patients, there was no difference in the rates of intubation or mortality at day 30 between HFNC (n = 44) and NIV (n = 44) [73] (Table 4). In a first prospective randomized non-inferiority clinical trial conducted in the emergency department, comparing HFNC and NIV in 204 patients with hypercapnic ARF from all causes, Doshi et al. [74] showed that HFNC was not inferior to NIV in terms of the risk of failure of the strategy (intubation or “switch”) applied within 72 h after randomization. The effect on PaCO_2_ changes during the first 4 h was also found to be comparable between HFNC and NIV groups, both in the overall population and in patients with hypercapnia at admission (Table 4). Beneficial results in terms of blood gas data, length of stay, and treatment success were also found to be similar between HFNC and NIV in a post-hoc analysis including only the AE-COPD subgroup from the previous study [75]. Cong et al. [76], who randomized 168 COPD patients, showed that HFNC and NIV were equally effective in improving arterial blood gas parameters after 12 h and 5 days of treatment. However, HFNC used at an average flow rate of 30−35 L/min was perceived as more comfortable than NIV and reduced the length of hospital stay. More recently, a prospective, randomized, multicenter non-inferiority study including 79 COPD patients with moderately severe hypercapnic ARF (pH = 7.25–7.35; PaCO_2_ ≥ 55 mmHg), initially treated with HFNC (n = 40) or NIV (n = 39), demonstrated that both strategies were equivalent in significantly reducing PaCO_2_ after 2 h and 6 h of use, with no difference in outcome (intubation, length of stay, and in-hospital mortality) [77]. However, 13/40 (32%) patients in the HFNC group had to change strategy and were switched to NIV after 6 h of treatment, which may have impacted the non-inferiority observed with HFNC. In 40 hypercapnic patients without respiratory acidosis at admission to the emergency department, a prospective randomized study showed that HFNC (n = 20) could further reduce PaCO_2_ in patients at hospital discharge compared to NIV (n = 20) [78]. Several systematic reviews and/or meta-analyses have evaluated previous data on the potential benefits of HFNC in hypercapnic ARF compared to NIV (Table 3). An initial study suggested that HFNC could generate interesting physiological effects (decrease or maintenance of PaCO_2_, comparable reduction in respiratory rate and work of breathing, improved comfort) similar to NIV [79]. More recently, several meta-analyses, including 5–8 randomized trials (528–735 patients) [64,65,80,81], concluded that, during the initial management of hypercapnic ARF (mainly AE-COPD) and compared to NIV, in addition to the absence of difference between the two strategies in improving respiratory parameters (dyspnea, respiratory rate) and in terms of patient outcomes (intubation, mortality, length of stay), HFNC was found to be more comfortable but required more switches to NIV for the most severe hypercapnic ARF (pH < 7.30) [64,65]. However, due to the heterogeneity of the studies and the lack of scientific evidence, further large-scale randomized studies were still needed to better determine the role of HFNC versus NIV in the management of hypercapnic ARF according to its severity and different underlying etiologies. Several randomized controlled trials were then published very recently (Table 4). In an initial single-center non-inferiority study, Tan et al. [82] found a higher failure rate (intubation and/or switch of treatment arm) in the HFNC group (n = 113 patients) than in the NIV group (n = 112) in patients with moderately severe COPD (25.7% vs. 14.3%, respectively; p = 0.033). Conversely, in another multicenter non-inferiority trial, Pantazopoulos et al. [83] reported that HFNC (n = 51) was as effective as NIV (n = 54) in preventing failure of the strategy (intubation and/or switch) in patients with mild to moderate AE-COPD. The latest non-inferiority study comparing HFNC to NIV in different situations of hypoxemic (immunocompromised or not, COVID-19, acute cardiogenic pulmonary edema (ACPE)) or hypercapnic ARF (AE-COPD with respiratory acidosis) was conducted in 33 Brazilian hospitals and included 1,800 patients from November 2019 to November 2023 [84]. For the subgroup of 79 AE-COPD patients randomized between HFNC (n = 35) and NIV (n = 42), no significant difference was found, i.e., HFNC was not inferior to NIV, with regard to the primary endpoint of intubation and/or death on day 7: 28.6% (10/35) vs. 26.2% (11/42), respectively; odds ratio = 1.05 (95% confidence interval (95% CI): 0.79–1.36). Nevertheless, given the small size of the AE-COPD subgroup and the somewhat sensitive results to the complex statistical model applied in this study, the authors recommended to perform additional, larger randomized studies in this population [84]. Finally, these last three randomized studies [[82], [83], [84]] were evaluated in a meta-analysis, including four randomized studies and a total of 486 patients, specifically comparing the initial management of AE-COPD with HFNC (n = 239 patients) or NIV (n = 247) [85] (Table 3). This meta-analysis reported no difference in all-cause mortality (relative risk (RR) = 0.97 (95% CI: 0.56–1.68)) or intubation rates (RR = 1.67 (95% CI: 0.99–2.83)) between the two groups. However, switch rates (RR = 2.60 (95% CI: 1.54–4.38)) and treatment failure rates (intubation and/or switch: (RR = 1.64 (95% CI: 1.04–2.60)) were significantly higher for patients in the HFNC groups. These results therefore confirm those of previous meta-analyses [64,65,80,81] (Table 3), namely that HFNC may be as effective as NIV for the initial management of hypercapnic ARF. In fact, the lower efficacy of HFNC compared to NIV could be partly masked by the more frequent switch in the strategy applied (changing from HFNC to NIV), particularly in patients with the most severe hypercapnic respiratory acidosis (pH < 7.30) [64,65]. In addition, the difference in effectiveness between HFNC and NIV must potentially take into account the presence or absence of associated obesity, which, in the case of underlying overlap syndrome, is likely to respond better to NIV than to HFNC [77,83].

**Table 4:** Main observationnal and randomized clinical studies using HFNC as an alternative to NIV in acute hypercapnic ARF.

Authors [reference] year	Country	Study design (global population)	Location	Study group(s) (n patients)	Initial settings	End-points (*primary)	Main results (with HFNC vs NIV)
Sun et al. [69] 2015	China	Single center Retrospective Observational (n = 82)	ICU	Hypercapnic AE-COPD (7.25 ≤ pH ≤ 7.35, PaCO_2_ > 50 mmHg) HFNC (n = 39) vs NIV (n = 43)	HFNC: Flow = 50 L/min FiO_2_/SpO_2_ target = 88−92% NIV: IPAP/EPAP = 10/4 cmH_2_O FiO_2_ for SpO_2_ = 88−92%	. Treament failure rate (intubation or switch to other group) * . 28-day mortality . Nursing interventions and device application (1^rst^ 24 h) . Skin breakdown	. No significant difference in treatment failure and mortality . Fewer nursing interventions (p < 0.05) and longer device application (p < 0.05) . Fewer skin breakdown (p < 0.05)
Su et al. [70] 2021	China	Single center Retrospective Observational (database) (n = 379) After propensity score matching (n = 112)	ICU	Mild hypercapnic ARF (45 mmHg < PaCO_2_ ≤ 60 mmHg) HFNC (n = 106) vs NIV (n = 106)	NR	. Outcome*: Intubation, mortality and ICU LOS . Performance of HR/SpO2 compared to SpO2/FiO2 (Rox index) to predict HFNC failure	. No significant difference in intubation rate at 48 h . Higher 28-day intubation rate (p = 0.029) and mortality (p = 0.043), longer ICU LOS (p = 0.019), . Better performance of HR/SpO2 to predict HFNC failure at 48 h (intubation, AUC: p < 0.01)
Rezaei et al. [71] 2021	Iran	Single center Prospective Randomized Cross-over (n = 30)	Respiratory ward	Mild to moderate hypercapnic AE-COPD (7.25 < pH < 7.35; PaCO_2_ ≥ 45 mmHg) HFNC followed by NIV (n = 15) NIV followed by HFNC (n = 15) each sequence = 30 min	HFNC: Flow = 20−30 L/min FiO_2_ = NR NIV: IPAP/EPAP/FiO_2_ = NR	. Changes in dyspnea score, RR, and ABG after 30 mn	. HFNC and NIV equally effective in the short term for improving ARF signs and ABG
Colaianni-Alfonso et al. [72] 2025	Argentina	Single center Retrospective Observational (n = 100)	ED RICU	Moderate hypercapnic AE-COPD (7.30 < pH < 7.35; PaCO_2_ ≥ 58 mmHg)	HFNC: Flow = 60 L/min FiO_2_ for SpO_2_ = 88−92% NIV: PS level for Vte = 6−8 mL/kg of IBW PEEP level = 5−10cmH_2_O	. Treament failure rate (intubation, switch to other group or death) * . Changes in RR and ABG, tolerance . LOS, duration of NIRS, 28-day mortality	. No significant difference in treatment failure rate, NIRS duration, hospital LOS and mortality . Inferior early improvement in RR (p = 0.015), and PaCO_2_ (p = 0.010) . Better tolerance (p < 0.001)
Lee et al. [73] 2018	Korea	Single center Prospective Observational (n = 88)	Respiratory ward	Moderate hypercapnic ARF (pH ≤ 7.35; PaCO_2_ ≥ 45 mmHg)	HFNC: Flow = 35 L/min (up to 45−60 L/mn) FiO_2_ for SpO_2_ ≥ 92% (n = 44) NIV: IPAP = 10 cmH_2_O EPAP = 5 cmH_2_O FiO_2_ for SpO_2_ ≥ 92% (n = 44)	. Intubation rate * . 30-day mortality rate . Changes in ABG at 6 and 24 h	. No significant difference in. intubation and 30-day mortality rates . No significant difference in ABG changes
Doshi et al. [74,75] 2018 et 2020	USA	Multicenter Prospective Randomized Non-inferiority (n = 204)	ED	Pre-defined post-hoc analysis: Hypercapnic ARF or AE-COPD (n = 65)	HFNC: Flow = 35 L/min FiO_2_ = 100% (n = 34) NIV: IPAP = 10 cmH_2_O EPAP = 5 cmH_2_O (n = 31)	. Changes in ABG (pH, PaCO_2_) * . Intubation and treament failure rate at 72 h (intubation, switch to other group)	. No significant difference in pH, PaCO_2_ changes . No significant difference in intubation and treatment failure rate
Cong et al. [76] 2019	China	Single center Prospective Randomized (n = 168)	ICU	Moderate to severe hypercapnic AE-COPD (pH = 7.25 ± 0.08; PaCO_2_ = 72 ± 16 mmHg)	HFNC: Flow = 30−35 L/min FiO_2_ for SpO_2_ = 88−92% (n = 84) NIV: IPAP = 10 cmH_2_O EPAP = 5 cmH_2_O (n = 84)	. Changes in ABG at 12 h and 5 days * . Complications and comfort . Hospital LOS	. No significant differences in ABG changes; . Lower complications . Better comfort and nursing satisfaction . Lower LOS
Cortegiani et al. [77] 2020	Italy	Multicenter Prospective Randomized Non-inferiority (n = 79)	ED ICU Respiratory ward	Mild to moderate hypercapnic AE-COPD (7.25 < pH < 7.35; PaCO_2_ ≥ 45 mmHg) HFNC (n = 40) vs NIV (n = 39)	HFNC: Flow = 60 L/min FiO_2_ for SpO_2_ = 88−92% NIV: PS level for Vte = 6−8 mL/kg of IBW PEEP level = 5 cmH_2_O FiO_2_ for SpO_2_ = 88−92%	. Changes in PaCO2 after 2 h* . Changes in PaCO2 and witch to other group within 6 h . Outcome (intubation, LOS, in-hospital mortality)	. Non inferior in reducing PaCO_2_ after 2 and 6 h . No significant difference in outcome . More patients (32%) requiring a switch to NIV (p = 0.0061)
Papachatzakis et al. [78] 2020	Greece	Single center Prospective Randomized (n = 40)	ED ICU	Mild to moderate hypercapnic ARF (PaCO_2_ ≥ 45 mmHg) HFNC (n = 20) vs NIV (n = 20)	HFNC: Flow = 35 L/min (up to 45−50 L/mn) FiO_2_ for SpO_2_ > 90% NIV: IPAP/EPAP gradually increased according tolerance FiO_2_ for SpO_2_ > 90%	. Changes in ABG (PaCO_2_)* . Changes in RR . Outcome (strategy duration, in hospital LOS, mortality)	. Further ↓ in PaCO_2_ at hospital discharge (p = 0.024) . Lower RR (p = 0.023) . No significant difference in outcome
Tan et al. [82] 2024	China	Single center Prospective Randomized Non-inferiority (n = 225)	ICU	Moderate hypercapnic AE-COPD (7.25 < pH < 7.35; PaCO_2_ ≥ 50 mmHg) HFNC (n = 113) vs NIV (n = 112)	HFNC: Flow = 40 L/min FiO_2_ for SpO_2_ = 88−92% NIV: IPAP/EPAP = 8/4 cmH_2_O gradually increased according tolerance FiO_2_ for SpO_2_ = 88−92%	. Treament failure rate (intubation and/or switch to other group) * . Changes in ABG, vital signs (HR, RR, BP) at 1,12 and 48 h . ICU and hospital LOS, 28-day mortality	. Higher failure rate (p = 0.033) . Higher intubation rate (p = 0.026) . No significant difference in ABG, vital signs changes except a higher RR (p = 0.025) and PaCO2 (*P* = 0.043) at 48 h . No significant difference in the switch rate, ICU and hospital LOS, 28-day mortality
Pantazopoulos et al. [83] 2024	Greece	Multicenter Prospective Randomized Non-inferiority (n = 105)	ED Respiratory ward	Mild to moderate hypercapnic AE-COPD (7.25 < pH < 7.35; PaCO_2_ > 45 mmHg) HFNC (n = 51) vs NIV (n = 54)	HFNC: Flow = 50−60 L/min FiO_2_ for SpO_2_ = 88−92% NIV: IPAP/EPAP = 15/3 cmH_2_O FiO_2_ for SpO_2_ = 88−92%	. Treatment failure rate (intubation and/or switch to other group) * . Changes in respiratory parameters (RR, ABG), dyspnea and respiratory comfort at different time points	. No significant difference in treatment failure . Lower dyspnea and comfort . No significant difference in respiratory parameters
Maia et al. [84] 2025	Brazil	Multicenter Prospective Randomized Non-inferiority (n = 1176)	ED ICU Hospital ward	Only mild to moderate hypercapnic AE-COPD (n = 79) (pH < 7.35; PaCO_2_ > 45 mmHg) HFNC (n = 37) vs NIV (n = 42)	HFNC: Flow = 40 L/min FiO_2_ for SpO_2_ = 88−92% NIV: IPAP/EPAP = 12−16/4 cmH_2_O FiO_2_ for SpO_2_ = 88−92%	. Intubation rate or death within 7 days * (Bayesian hierarchical model) . MV and ICU free-days at 28 days . 90-day ICU and hospital LOS . 28-day and 90-day mortality	. No significant difference in the primary outcome . No significant difference in other outcomes

HFNC: high-flow nasal cannula; ARF: acute respiratory failure; AE-COPD: acute exacerbation of chronic obstructive pulmonary disease; NIV: non invasive ventilation; NIRS: non invasive respiratory support; PS: pressure support; PEEP: positive end expiratory pressure; IPAP: inspiratory positive airway pressure; EPAP: expiratory positive airway pressure; RCT: randomized controlled trial; ICU: intensive care unit; ED: emergency department; RICU: respiratory intensive care unit; ABG: arterial blood gas; IBW: ideal body weight; NR: not reported; RR: respiratory rate; HR: heart rate; BP: arterial blood pressure; AUC: area under the receiver operating characteristic curve; LOS: length of stay.

Beyond AE-COPD, a preliminary observational study showed that HFNC also improved PaCO_2_ and respiratory parameters similarly to NIV from the first hour of treatment in patients with hypercapnic ACPE admitted to the emergency department [86]. In this context, the absence of difference between the two strategies in terms of respiratory rate, hematosis, and patient outcomes was confirmed by a recent randomized multicenter pilot study conducted in 60 patients by the same team [87]. Finally, a meta-analysis evaluating six randomized or observational prospective or retrospective studies, including the two previous ones, with a total of 572 patients (221 HFNC vs. 351 NIV), showed that, compared to NIV, HFNC did not increase the risk of treatment failure (early discontinuation, intubation, and/or death) during the initial management of suspected or confirmed ACPE [88].

##### High-flow nasal cannula therapy in combination with non-invasive ventilation

In addition to the use of HFNC alone as an alternative to NIV as first-line treatment during hypercapnic ARF, the operating principles and physiological effects of HFNC [[5], [6], [7], [8]] also allow for its clinical use in combination with NIV sessions during spontaneous ventilation phases instead of standard oxygen therapy. As demonstrated during hypoxemic ARF [37], the sequential use of HFNC with NIV (NIV + HFNC) during AE-COPD could therefore, in addition to maintaining satisfactory gas exchange (pH, PaCO_2_) compared to the NIV period, also reduce respiratory rate and work of breathing while improving patients' respiratory comfort, compared to conventional oxygen therapy alternated with NIV (NIV + O_2_) [21]. Although used in clinical practice in cases of COPD exacerbation at admission by some teams [89], few studies have evaluated this combined ventilation strategy.

A retrospective, single-center cohort study including 351 COPD patients with acute exacerbation requiring NIV (pH < 7.35) showed no additional benefit in terms of intubation or 30-day mortality of the combination of NIV and HFNC (n = 126) compared to a standard strategy combining NIV with standard oxygen [90]. To our knowledge, only one prospective, multicenter, randomized study has evaluated the clinical benefit of HFNC compared to standard oxygen as supplemental respiratory support during periods of NIV interruption [91]. In 47 patients with hypercapnic ARF (pH < 7.35, PaCO_2_>45 mmHg), 62% of whom had COPD, the authors found no difference in the time spent on NIV or spontaneous ventilation (HFNC or standard oxygen) between the two strategies, but compared to NIV, HFNC further reduced dyspnea and respiratory rate and proved to be more comfortable for patients.

Like other authors [92], we believe that an association of NIV and HFNC could be the most useful and relevant strategy in the most severe forms of ARF with hypercapnic acidosis in COPD patients, in particular to avoid intubation and improve patient morbidity and mortality. The results of ongoing prospective randomized trials seeking to evaluate the physiological and/or clinical benefits of a strategy combining NIV + HFNC compared to the standard strategy (NIV + O_2_) should confirm this hypothesis [93,94]. However, it should be kept in mind that, apart from COPD, HFNC has not been specifically evaluated in hypercapnic ARF due to other etiologies such as obesity hypoventilation syndrome or even neuromuscular disorders.

#### Management of the post-extubation period

After intubation for ARF, weaning/extubation from invasive mechanical ventilation is a real daily challenge for critical care clinicians. Indeed, the risk of failure with reintubation due to post-extubation ARF can be observed in 10–20% of patients who have undergone planned extubation and has long been recognized as an independent risk factor of morbidity and mortality [95]. Although the association of NIV with HFNC is currently the recommended strategy for preventing post-extubation ARF in patients at risk of reintubation [96], HFNC alone could potentially be an interesting alternative to overcome the disadvantages of NIV (intolerance and discomfort) and prevent this risk, particularly in hypercapnic patients during extubation.

A retrospective observational study, conducted in China in 157 extubated COPD patients (PaCO_2_ ≥ 50 mmHg before extubation), showed no difference in reintubation rates within 72 h, length of stay in respiratory intensive care, or in-hospital mortality between the use of HFNC (n = 114) or NIV (n = 143) post-extubation [97]. However, HFNC was found to be more beneficial (lower reintubation rate and shorter length of stay) in the subgroup of patients with the highest secretions (sputum volume ≥ 20 mL within 24 h post-extubation). An initial randomized pilot study reported no difference between HFNC (n = 20) and NIV (n = 20) in terms of blood gas data and vital signs, duration of invasive mechanical ventilation, reintubation rate, RICU length of stay, and mortality at day 28 in 40 COPD patients who remained hypercapnic after extubation [98]. It should be noted that, in this study, despite improved respiratory comfort, HFNC also reduced the need for bronchoscopy within 48 h post-extubation. A prospective, randomized, multicenter study also showed that HFNC (n = 44) was not inferior to NIV (n = 42) in preventing treatment failure (reintubation or switch between the two strategies) in the post-extubation period in COPD patients initially intubated for hypercapnic ARF [99]. Finally, a meta-analysis focused specifically on the management of AE-COPD after extubation [100]. Including eight randomized studies, four of which were in Chinese language, and a total of 612 patients, this meta-analysis showed that HFNC could be just as effective as NIV in terms of preventing reintubation, ICU length of stay and mortality, especially in hypercapnic patients, with potentially fewer complications (intolerance to the technique, skin lesions) than with NIV. These meta-analytic data were supported by a recent prospective randomized single-center study showing comparable efficacy of HFNC (n = 30) and NIV (n = 32) in reducing extubation failure (reintubation or switch of strategy) with no difference in length of stay, and HFNC was also found to be better tolerated than NIV [101]. However, as shown in the post-hoc analysis of the large multicenter randomized trial “High-Wean” [96], the clinical benefit of reducing the risk of post-extubation ARF and reintubation appears to be greater when combining, in the post-extubation period in COPD patients, HFNC alternating with prophylactic NIV (n = 86) compared to HFNC alone (n = 64), although there was no impact on ICU mortality between the two strategies [102]. While it is likely that HFNC and NIV may have a comparable effect in preventing the risk of post-extubation ARF and reintubation in patients at the lowest risk of extubation failure, pending the results of further studies [103], the combination of NIV + HFNC currently appears to be the most appropriate strategy in patients at the highest risk of extubation failure, particularly in hypercapnic COPD patients [92,104].

## Conclusion

Although NIV remains the first-line ventilatory strategy in the management of hypercapnic ARF [47,105], the operating principles and physiological effects of HFNC suggest that it could be used in this indication, either in the initial phase and/or following extubation, particularly in COPD patients. Indeed, the clinical data currently available in mild to moderate hypercapnic ARF, comparing HFNC and standard oxygen therapy or NIV, although limited in terms of scientific evidence, appear very promising in this regard. However, additional prospective randomized studies are still needed to better define the role of HFNC in hypercapnic ARF, particularly in patients with the most severe acidosis [106]. Pending the results of ongoing or future randomized clinical trials [93,94,103,107] to optimize the individualization of noninvasive respiratory support strategies, it is likely that the combination of NIV and HFNC (NIV + HFNC), taking into account NIV tolerance, could be the most useful and relevant strategy for this specific population.

## CRediT authorship contribution statement

• Substantial contributions to the conception or design of the work: C.G. and E.A.M.
• Acquisition, analysis, or interpretation of data for the work: C.G., E.A.M. and P.L. D.
• Drafting the work or revising it critically for important intellectual content: C.G., P.L.D., J.P.F., J.D.R., A.W.T and G.B.
• Final approval of the version submitted for publication: all authors.

## Consent for publication

Not applicable.

## Ethics approval and consent to participate

Not applicable.

## Funding

Not applicable.

## Availability of data and materials

The datasets used and/or analysed during the current study are available from the corresponding author on reasonable request.

## Declaration of competing interest

C.G. declare support for attending meetings and/or travel, grants and non financial support by Fischer & Paykel Healthcare and Asten Santé; Equipment and materials for education and training by Fischer & Paykel Healthcare, Resmed, Lowenstein Medical and Air Liquide.

E.A.M declare support for attending meetings and/or travel, grants and non financial support by Fischer & Paykel Healthcare, Asten Santé, GEP and Epione Santé; Equipment and materials for education and training by Fischer & Paykel Healthcare.

P.L.D declare support for attending meetings and/or travel, equipment and materials for education and training by Fischer & Paykel Healthcare.

J.P.F. has received personal fees for lecture, and travel expense coverage to attend scientific meetings from Fisher & Paykel Healthcare; and grant for studies, personal fees as a member of a scientific board, travel expense coverage to attend scientific meetings from SOS oxygène.

J.D.R. has received fees for lecture, and travel expense coverage to attend scientific meetings from Fisher & Paykel Healthcare; and grants from the French ministry of Health.

A.W.T. declare grants from the French ministry of Health, personal fees (payment for lectures, and travel/ accommodation expense coverage to attend scientific meetings) and non-financial support from Fisher&Paykel Healthcare.

G.B. declare that he as no competing interest.
